# Supplementary figures and images for: OrganoID: A versatile deep learning platform for tracking and analysis of single-organoid dynamics
Source: PLoS Comput Biol. 2022 Nov 9;18(11):e1010584. doi: 10.1371/journal.pcbi.1010584 (PMC9645660; doi:10.1371/journal.pcbi.1010584)

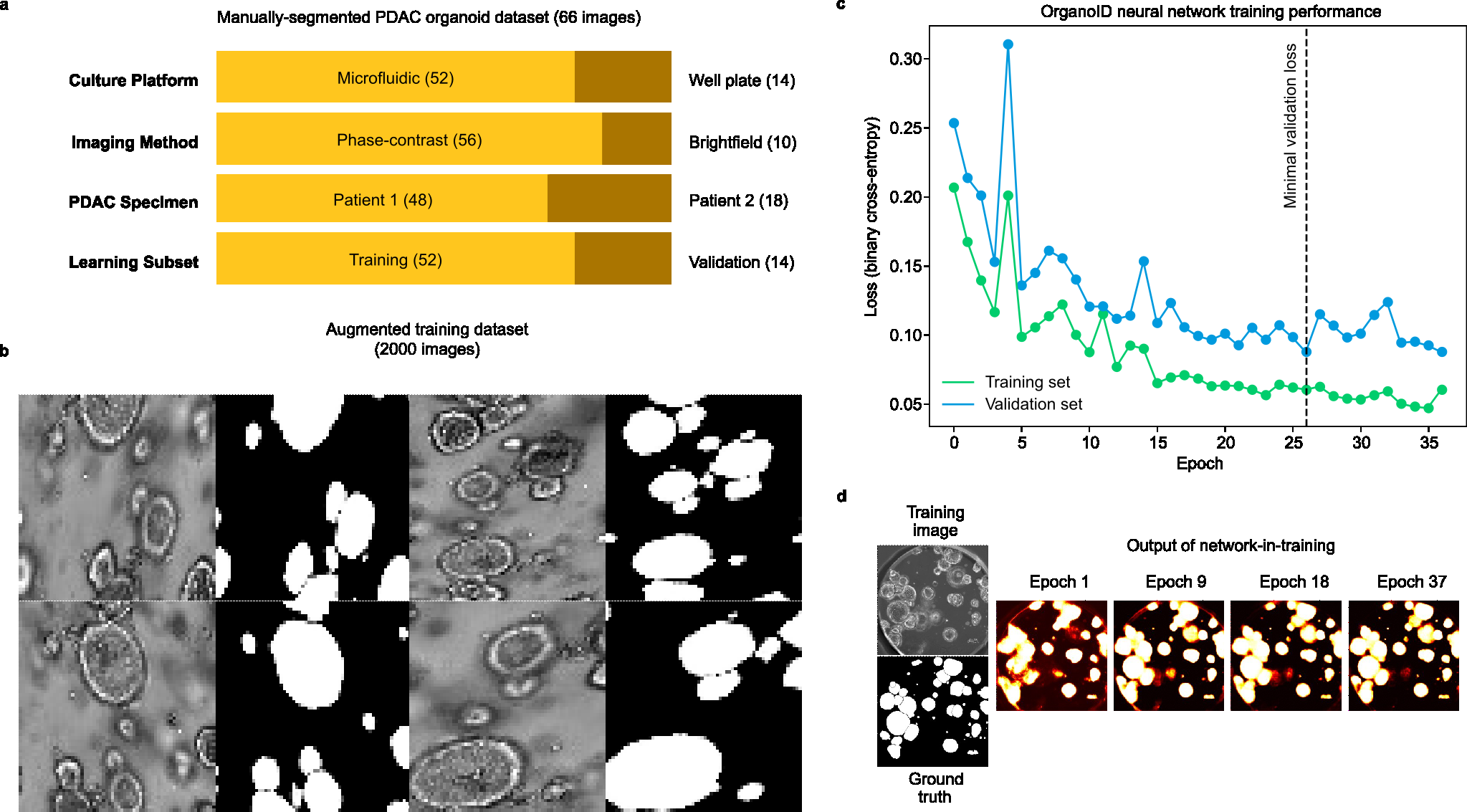

Supplement: S1 Fig — (a) The network was trained on 66 manually labeled microscopy images of organoids derived from pancreatic ductal adenocarcinoma (PDAC) samples from two patients. Organoids were cultured in a well plate or microfluidic format and imaged through phase-contrast or brightfield microscopy. Images were then split into datasets for network training (80%) and validation (20%). (b) The 52 images in the training dataset were passed through a series of random transformations to produce an augmented dataset of 2,000 images. (c) Network training was stopped after 37 epochs, once a minimum binary cross-entropy loss on the validation dataset was reached. (d) The OrganoID neural network predicts the probability that an organoid is present at each pixel. Shown are network predictions produced by intermediate models at selected epochs through the training process. (TIF) [file pcbi.1010584.s001.tif]

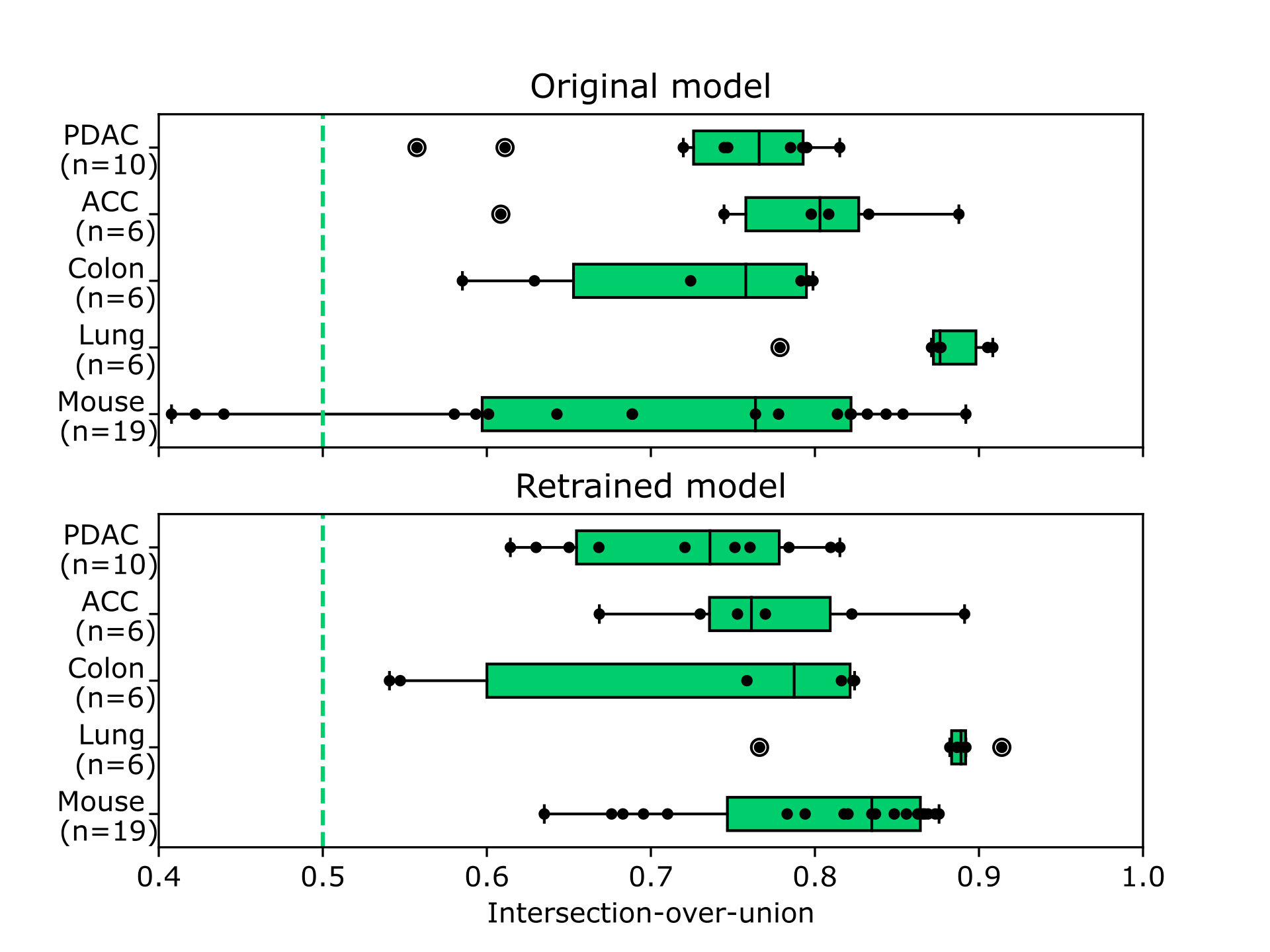

Supplement: S2 Fig — A test set of images of organoids derived from human PDAC, salivary adenoid cystic carcinoma (ACC), colon epithelia, distal lung epithelia, and mouse small intestine were manually segmented to assess network performance (top). An IOU of 0.5 was set as a benchmark for a successful network prediction (dashed green line). All images of human organoids in the test set passed the benchmark, which demonstrates the capacity of the PDAC-trained network to generalize to other organoid types. Several of the mouse organoids did not pass the benchmark, and so the model was later retrained with part of this dataset included to demonstrate extensibility (bottom). (TIF) [file pcbi.1010584.s002.tif]

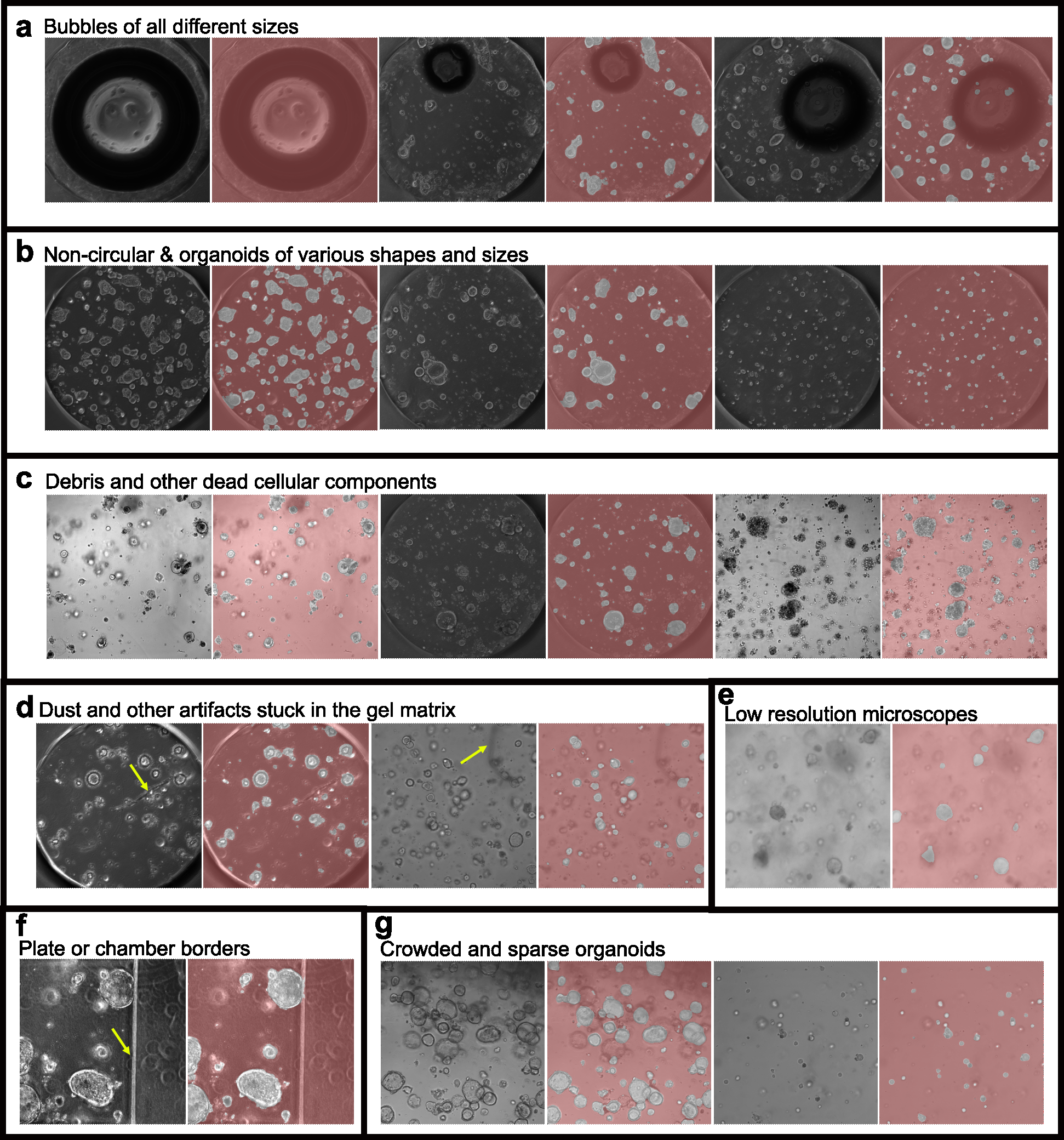

Supplement: S3 Fig — OrganoID ignored bubbles (a), debris (c-d), and plate or microfluidic chamber borders (f) to accurately identify organoids that exhibit diverse morphology and sizes, even within a single sample (b). OrganoID can also handle various optical configurations, including low-resolution or poorly-lit images (e). Gel droplets can support densely-packed or isolated organoids, which can all be detected with OrganoID (g). (TIF) [file pcbi.1010584.s003.tif]

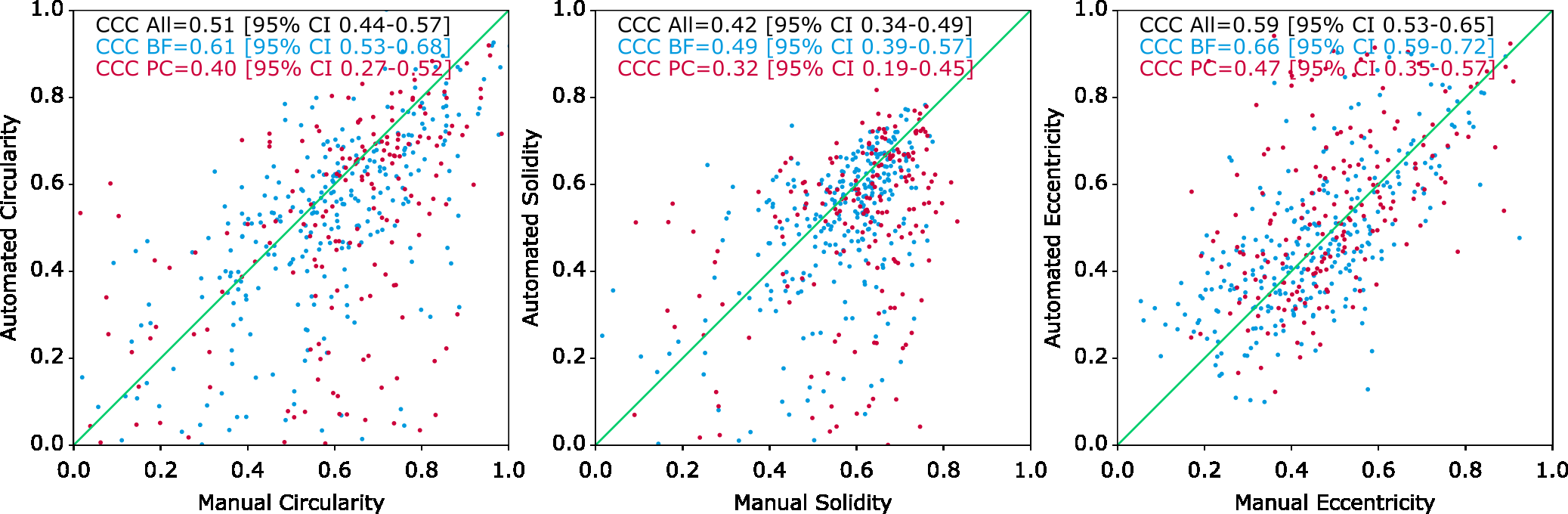

Supplement: S4 Fig — OrganoID was used to measure organoid circularity (ratio of organoid area to the area of a perfect circle with equal perimeter), solidity (ratio of organoid area to area of the convex hull), and eccentricity (elliptical deviation from a circle). These measurements were then compared to those from manual segmentation. The concordance correlation coefficient (CCC) was computed for all organoids, as well as for organoids imaged through phase contrast (PC, red) or brightfield (BF, blue) microscopy. For calculation of CCC for circularity and solidity (bounded to 0–1, with most values near 1), the data was first logit-transformed. (TIF) [file pcbi.1010584.s004.tif]

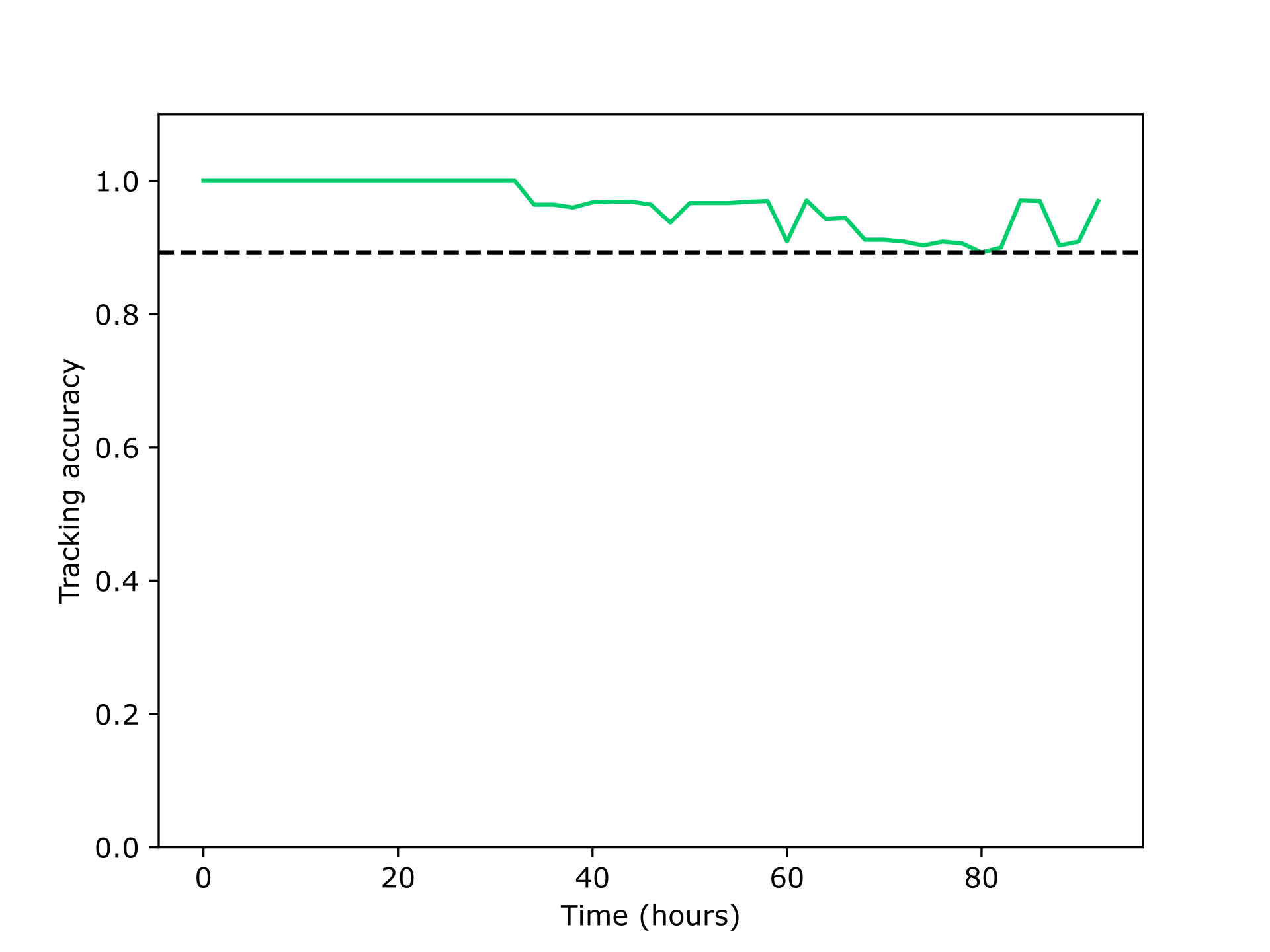

Supplement: S5 Fig — A time-lapse microscopy experiment was analyzed with OrganoID to identify organoids in each image. OrganoID was then used to match identified organoids across frames to build single-organoid tracks. The identified organoids were also matched by hand to assess tracking performance. Accuracy was defined the number of organoid track labels in agreement divided by the total number of organoids present at each frame. (TIF) [file pcbi.1010584.s005.tif]

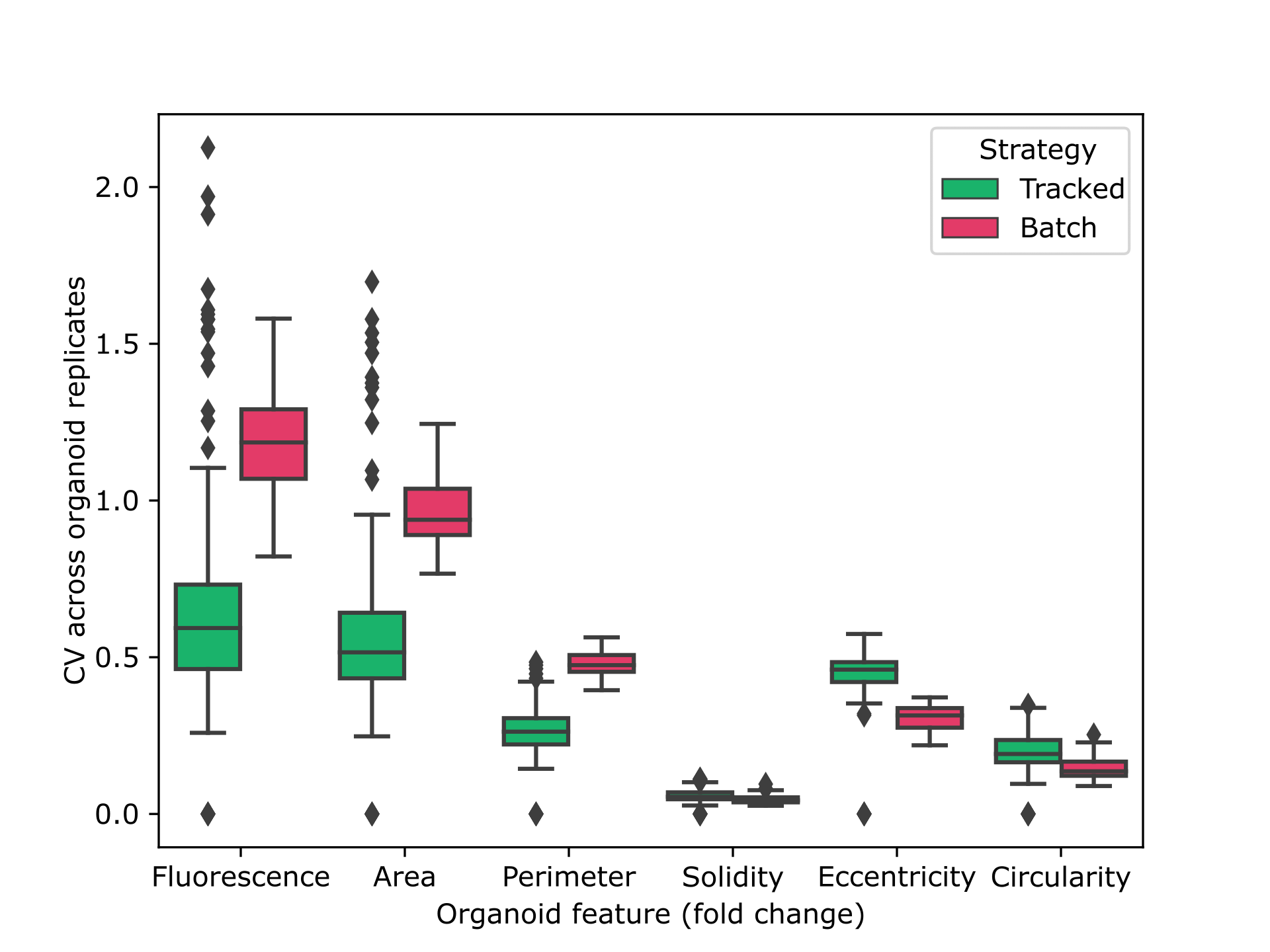

Supplement: S6 Fig — In batch analysis, measurements of organoids exposed to the same gemcitabine dosage for the same duration were normalized to the average of organoids at t = 0. In tracked analysis, organoid measurements are instead normalized to each individual organoid measurement when initially detected by the tracking algorithm. The coefficient of variation (CV) was significantly lower with tracked analysis for change in fluorescence (Welch’s t-test p = 2e-30), area (p = 2e-29) and perimeter (2e-63). CV was significantly higher with tracked analysis for eccentricity (p = 7e-25) and circularity (p = 2e-12). (TIF) [file pcbi.1010584.s006.tif]

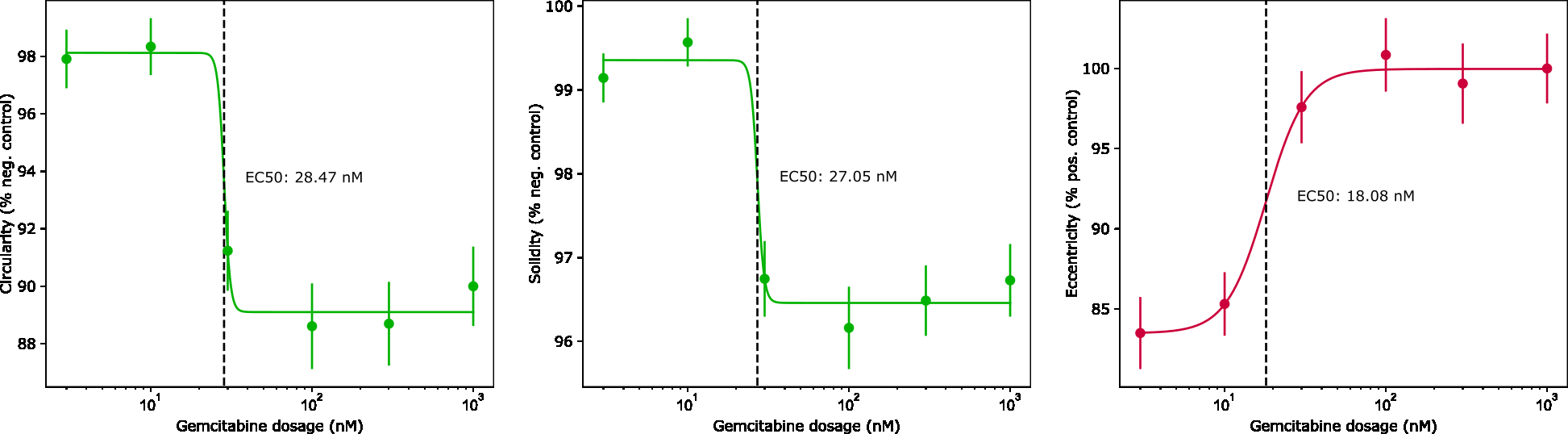

Supplement: S7 Fig — Organoid circularity, solidity, and eccentricity were observed to follow sigmoidal dose responses to gemcitabine. (TIF) [file pcbi.1010584.s007.tif]

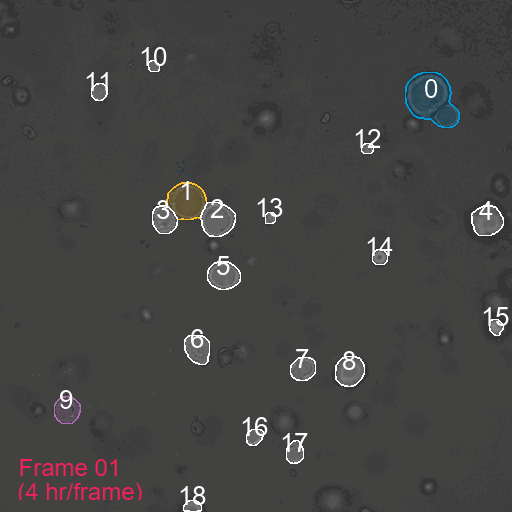

Supplement: S2 Video — (GIF) [file pcbi.1010584.s009.gif]
