# Supplementary material for: OrganoID: A versatile deep learning platform for tracking and analysis of single-organoid dynamics
Source: PLoS Comput Biol. 2022 Nov 9;18(11):e1010584. doi: 10.1371/journal.pcbi.1010584 (PMC9645660; doi:10.1371/journal.pcbi.1010584)
Supplement: S1 Methods — (DOCX) [file pcbi.1010584.s011.docx]

# Supplementary Methods

**Organoid culture and image acquisition**

Tumor organoid cultures derived from pancreatic adenocarcinoma (PDAC) patients were isolated and prepared as previously described [1]. PDAC organoids were cultured and imaged in a microfluidic platform [2] or a 24-well suspension culture plate (Thermo Fisher, 144530). Human colonic organoids were cultured similarly to PDAC organoids, with Matrigel and growth or differentiation media as described in established methods [1]. Distal respiratory organoids were obtained from the Ishay-Ronen Lab at the Sheba Medical Center and cultured through a previously described protocol [3] in 24-well plates. Images of adenoid cystic carcinoma (ACC) organoids and mouse small intestinal organoids were obtained from Dr. Weber and colleagues from University of Chicago Medicine. The pancreatic and airway lung organoids were cultured and imaged on an automated translational stage of an inverted microscope (Nikon Eclipse Ti) enclosed in an environmentally controlled chamber (Life Imaging Service GmbH, Basel, Switzerland). The enclosure provides temperature, humidity, and CO2 gas control to maintain adequate cell culture conditions for the organoids. Organoids were cultured at a constant 37°C, 5% CO2, a humidity flow rate of 25-30 L/hour, and 95-100% relative humidity. Images of the organoids were acquired through the standard microscope software that is capable of automatically acquiring images at different positions, *Z*-planes/stacks, and multiple fluorescent filters (NIS-Elements software, Japan). The microscope was equipped with a digital complementary metal-oxide semiconductor (CMOS) camera (ORCA-Flash 4.0, Hamamatsu, Japan), which imaged the organoids using a x10 objective at 2 to 4-hour intervals.

**Dataset for evaluation of network performance and generalizability**

The network structure and training process were manually tuned for performance on the PDAC validation dataset. After optimization, we locked out any further changes to all hyperparameters and evaluated final network performance and generalizability on a separate dataset of 28 organoid microscopy images. The dataset included images of organoids derived from PDAC (10), benign colon tumor cells (6), lung epithelia (6), and salivary adenoid cystic carcinoma (6). The images were obtained through brightfield and phase-contrast microscopy from multiple microscopy cores and manually labeled by two independent reviewers.

**Network evaluation and retraining on mouse small intestine organoids**

19 images of organoids derived from mouse small intestine were obtained and manually segmented. The OrganoID platform was used to segment and identify individual organoids in these images, which were compared to the manually segmented images. For network retraining, the mouse organoid images were randomly split into groups for training (11), validation (4), and testing (4). The training image set was augmented as above to produce 500 images, which were appended to the original training dataset. The OrganoID neural network was then retrained on this expanded training dataset for an additional 20 epochs and evaluated for performance on the test mouse organoid images as well as the original testing images.

**Statistical methods for platform validation**

Statistical analysis was performed with the *numpy* and *scipy* packages in Python. Network performance on the testing dataset was evaluated as the pixel-wise intersection-over-union (IOU) of predictions compared to ground-truth segmentations. A single IOU value was computed for each prediction/ground-truth pair and summarized as a mean IOU and standard deviation over the dataset. For agreement of single-organoid counts and measurements, the Lin concordance correlation coefficient (CCC) was computed as:

$$CCC=\frac{2s_{xy}}{{s_{x}}^{2}+{s_{y}}^{2}+(\bar{x} -\bar{y})^{2}}$$

The Fisher transformation ($arctanh(CCC)$) and the inverse Fisher transformation ($tanh(z_{lo}, z_{hi})$) were also used to obtain a z-value and construct a 95% confidence interval for CCC statistics. Effect sizes were computed with Cohen’s d statistic ($2(M_{1}-M_{2})/\sqrt{\sigma_{1}^{2}+\sigma_{2}^{2}}$). All plots were generated with the *matplotlib* and *seaborn* packages in Python.

**Drug screening experiments**

PDAC organoids were grown for one week in standard culture conditions and then treated with gemcitabine hydrochloride (G6423, Sigma) at six serial dilutions from 3 nM to 1000 nM with negative control. Propidium iodide (Thermo Fisher P3566) was used to fluorescently measure cellular death and relative viability of organoids in real-time at 4-hour intervals over 72 hours. An MTS proliferation assay (Promega, G3580) was also performed at the end of the experiment to determine cell viability for each condition according to manufacturer’s instructions. Statistical hypothesis testing was performed with *scipy* and MATLAB. Organoid circularity was computed as the circumference of a circle with equivalent area divided by the actual perimeter of the organoid.

**Validation rigor and indications of robustness**

The OrganoID neural network was tested on images of four types of organoids taken at multiple microscopy cores. Images were manually segmented by two independent evaluators. OrganoID single-organoid count and area measurements were compared against the corresponding measurements of the manual test segmentations. OrganoID tracking results were compared to manually tracked images. The OrganoID platform and test microscopy images were distributed to an independent user to confirm reproducibility of all metrics, statistics, and figures.

# References

1. Romero-Calvo I, Weber CR, Ray M, Brown M, Kirby K, Nandi RK, et al. Human Organoids Share Structural and Genetic Features with Primary Pancreatic Adenocarcinoma Tumors. Mol Cancer Res [Internet]. 2018/08/31 ed. 2019 Jan;17(1):70–83. Available from: https://pubmed.ncbi.nlm.nih.gov/30171177

2. Schuster B, Junkin M, Kashaf SS, Romero-Calvo I, Kirby K, Matthews J, et al. Automated microfluidic platform for dynamic and combinatorial drug screening of tumor organoids. Nat Commun [Internet]. 2020 Oct 19;11(1):5271–5271. Available from: https://pubmed.ncbi.nlm.nih.gov/33077832

3. Sachs N, Papaspyropoulos A, Zomer-van Ommen DD, Heo I, Böttinger L, Klay D, et al. Long-term expanding human airway organoids for disease modeling. EMBO J [Internet]. 2019/01/14 ed. 2019 Feb 15;38(4):e100300. Available from: https://pubmed.ncbi.nlm.nih.gov/30643021
