# Supplementary material for: OrganoID: A versatile deep learning platform for tracking and analysis of single-organoid dynamics
Source: PLoS Comput Biol. 2022 Nov 9;18(11):e1010584. doi: 10.1371/journal.pcbi.1010584 (PMC9645660; doi:10.1371/journal.pcbi.1010584)
Supplement: Attachment — Submitted filename: PLOS ONE ORGANOID Review.docx [file pcbi.1010584.s012.docx]

Review:

**Title: OrganoID: a versatile deep learning platform for tracking and analysis of single- organoid dynamics**

Authors: Jonathan Matthews, Brooke Schuster, Sara Saheb Kashaf, Ping Liu, Rakefet Ben- Yishay, Dana Ishay-Ronen, Le Shen, Christopher Weber, Margaret Bielski, Sonia S. Kupfer, Mustafa Bilgic, Andrey Rzhetsky, Savaş Tay

**Confidential comments to the editor**

In this manuscript, the authors present their findings of a machine-learning-based image analysis model, OrganoID. The authors have developed a novel platform in OrganoID that can be used to monitor drug responses by tracking organoid dynamics via deep learning. The authors have emphasized the importance of studying organoids, highlighted the limitations of the existing approach, and clearly stated the motivation for an automated image analysis tool. Furthermore, the greatest strength of this model is that it can be applied to a variety of tissues. Overall the paper is very well written especially the results section which has been broken down under further subheadings to make the paper easily comprehensible. A few revisions and clarifications are recommended to make the paper more robust.

**Confidential comments to the author**

In this manuscript, the authors present their findings of a machine-learning-based image analysis model, OrganoID. The authors have developed a novel platform in OrganoID that can be used to monitor drug responses by tracking organoid dynamics via deep learning. The authors have emphasized the importance of studying organoids, highlighted the limitations of the existing approach, and clearly stated the motivation for an automated image analysis tool. Furthermore, the greatest strength of this model is that it can be applied to a variety of tissues. Overall the paper is very well written especially the results section which has been broken down under further subheadings to make the paper easily comprehensible. A few revisions and clarifications are recommended to make the paper more robust.

**Major Revisions**

**Introduction**

**“**Three-dimensional (3D) cell culture systems serve as more physiologically relevant *in vitro* models than traditional human cell monolayers for both basic and clinical applications” (Why is that so? It is suggested that the authors further elaborate and expand on this claim. Furthermore, what do the authors mean by the phrase “physiologically relevant” - the reference cited states that organoid provides a powerful means for “organogenesis” and “tissue morphogenesis” – is that what the authors are referring to?)

“Live-cell imaging can reveal important features of organoid dynamics, such as growth, apoptosis/necrosis, and movement, which can reflect physiological and pathological behaviors and drug responses.” (Please cite a reference for this statement. What are authors referring to specifically when they are talking about “physiological and pathological behaviors”?)

“While organoids have been successfully used to investigate important phenomena that might be obscured in simpler models, their use in data-intensive applications, such as high-throughput screening, has been more difficult.” (Please cite a reference for this claim. Furthermore, it is suggested that the authors expand further on why it has been more difficult)

**Results**

“The OrganoID neural network follows a *u-net* structure and was optimized to require far fewer feature channels than the original implementation.” (It is suggested that the authors explain why they decided to optimize it to require fewer channels for clarity)

“Model simplification can limit overfitting and minimizes the amount of memory and computational power required to use the network in the final distribution.” (Is this statement made by the authors based on the results of prior study if yes, please cite the relevant study; however, if this is based on the authors' experiences it is suggested that they explain this further.)

“Additionally, all hidden convolutional layers were set to compute outputs with the exponential linear unit (ELU) activation function^26^, which has demonstrated higher accuracy than the rectified linear unit (ReLU) and avoids vanishing gradient problems during network training.” (Did it demonstrate a higher accuracy during this specific study’s network training or during another study – please elaborate and add specific statistics to highlight the “higher accuracy”. It would be helpful to back the replacement of ELU with ReLU with IOU scores)

“18 microscopy images of organoids derived from lung epithelia, colon epithelia, and salivary adenoid cystic carcinoma (ACC) were manually segmented.” (Were they 18 images from each tissue or 18 combined? If 18 combined please highlight how many images were used from each tissue. Why were such few images available?)

“The OrganoID network passed the benchmark for all non-PDAC images, with a mean IOU of 0.79 (SD=0.096). These results demonstrate the capability of the OrganoID neural network to reliably detect organoids from various tissues of origin, from both malignant and non-malignant tissues, in a pixel-by-pixel manner “(An IOU greater than 0.5 definitely demonstrates the capability of the model but perhaps saying the results are “reliable” without any statistical test to back it up is misleading. Given the small sample size may be the authors should revise this statement. In the introduction section, the authors have shared the relevant limitations of existing approaches in the literature. However, if feasible, it will be helpful to report the performance (IOU) of existing/ old approaches for at least semantic segmentation tasks to serve as a good reference point for comparing the proposed architecture.)

“For quantitative validation, OrganoID was used to count and measure the area of organoids in images from the PDAC and non-PDAC testing datasets (a total of 28 images).” (Why was the analysis not conducted on a larger image dataset?)

“Overall, the measurements produced by OrganoID were in considerable agreement with those obtained by hand, which supports the use of OrganoID for automated single-organoid analysis.” (Yes, the results demonstrate considerable agreement between the two however, the image dataset size is too small to claim that the results support the use of OrganoID for automated single-organoid analysis. Please revise this statement perhaps instead to state that it demonstrates that the model is capable of identifying organoids when compared to hand however, to demonstrate its utility it needs to be trained on more data.)

“Identification of individual organoids with diverse morphology and size” (The edge prediction depends on the labeling protocol used for the data with a thin boundary between organoids. This might not be true for another organoid dataset. It will be helpful if the authors can add some points for expanding this approach to a new dataset.)

The tracking step was also performed by hand to evaluate automated performance, which maintained over 89% accuracy throughout the duration of the experiment (What statistical test was used to measure this accuracy?)

Each organoid shape was then used to calculate the organoid circularity, a morphological measurement that compares the organoid perimeter to the perimeter of a perfect circle. (Given the morphology of the cells it is understood why this parameter was used but perhaps the authors should go into more detail about why they picked organoid circularity and why they think it is related to overall cellular architecture.)

**Discussion**

“Experimental replicates in organoid studies can differ in the number and distribution of sizes of organoids.” (Given these circumstances how would the authors suggest validating their model?)

“OrganoID achieves comprehensive and expedient image analysis of organoid experiments to enable the broader use of organoids as tissue models for high-throughput investigations into physiological and pathological phenomena.” (What do the authors mean by physiological and pathological phenomena, please elaborate.)

**Methods**

The methods section mentions that the appropriate number of filters in the first layer has been determined using validation set scores. It will be helpful if the authors can report those numbers.

**Minor Revisions**

**Abstract**

“Dynamic changes in individual organoid morphology, number, and size can indicate important drug responses, however these metrics are difficult and labor-intensive to obtain for high-throughput image datasets.” (please add a comma after however)

**Introduction**

**“**Three-dimensional (3D) cell culture systems serve as more physiologically relevant *in vitro* models than traditional human cell monolayers for both basic and clinical applications^1^. Organoids are multicellular 3D structures derived from primary or stem cells that are embedded into a biological matrix to create an extracellular environment that provides structural support and key growth factors.” (The reviewer suggests that the paragraph should be rephrased such that the concept of organoids is introduced before talking about its basic and clinical applications i.e. “Organoids are multicellular 3D structures derived from primary or stem cells that are embedded into a biological matrix to create an extracellular environment that provides structural support and key growth factors. Three-dimensional (3D) cell culture systems serve as more physiologically relevant *in vitro* models than traditional human cell monolayers for both basic and clinical applications”)

Many of these existing platforms were also developed for analysis of organoids derived from a single type of tissue and for images obtained with one specific geometric configuration, precluding their use across different experimental settings. (“the” missing – many of these existing platforms were also developed for “the” analysis of organoids)

**Results**

“The image is further refined to remove organoids that may be partially out of the field-of-view or are below a particular size threshold.” (The dashes between “field-of-view”are not needed)

“We also compared these normalized measurements to normalization with an MTS proliferation assay, which can only be used for endpoint analysis.” (what does MTS stand for?)

**Discussion**

**“…**express fluorescent proteins, however this increases experimental time and complexity and may alter cellular dynamics from the original tissue sample.” (add a comma after however)

“OrganoID was trained with and tested on a diverse, but relatively small set of images.” (“a” is missing before relatively)
