# Supplementary material for: OrganoID: A versatile deep learning platform for tracking and analysis of single-organoid dynamics
Source: PLoS Comput Biol. 2022 Nov 9;18(11):e1010584. doi: 10.1371/journal.pcbi.1010584 (PMC9645660; doi:10.1371/journal.pcbi.1010584)
Supplement: Attachment — Submitted filename: organoID_reviewer_response.docx [file pcbi.1010584.s013.docx]

**SPECIFIC RESPONSES TO REVIEWER COMMENTS**

Reviewer 1

High-confidence and robust methods for the analysis of easy-to-obtain brightfield images of organoid cultures are a central bottleneck for the high-throughput processing of clinically relevant in vitro drug-response screens and thus a timely problem. Matthews and colleagues present a methodology to segment, track and analyze organoid shape from brightfield and phase-contrast microscopy images that can be potentially used for the high-throughput analysis of organoid screens. The heart of the analysis routine which they call OrganoID is a u-net-based neuronal network that was trained to segment organoids using a training data set of originally 66 images of pancreatic ductal adenocarcinoma (PDAC). The training data set was computationally increased by multiple rigid and elastic image transformations. The accuracy of the segmentation was compared to manually segmented ground-truth models and revealed a high IOU (0.74) for PDAC and of 0.79 for organoids from other tissues such as lung and colon as well as a low false positive rate (4.2%). To segment individual organoids the authors followed a classical Canny edge detection followed by watershed segmentation procedure. The number of automatically detected organoids was very close to manually identified organoids. Individual organoids were tracked over time with an estimated accuracy of 89%. Next the authors show a case study to quantify the response of PDAC organoids to the chemotherapeutic drug gemcitabine. Based on the segmented organoid masks they measure PI intensity as indicator for cell death and see a decrease in organoid area accompanied by an increase of PI with increasing drug concentration. Finally, the authors take circularity as one shape parameter and claim that it is predictive for a drug response.

**RESPONSE 1: We thank the reviewer for recognizing the importance of the problem of organoid image analysis and concisely summarizing our approach and results.**

The presented analysis procedure works well on the test data set, and altogether appears to be promising. It is difficult, though, to really judge the robustness of the method due to the low number of test images. The authors themselves admit that the method was trained and tested on a small data set (66 PDAC and 18 other lines)– therefore it is difficult to argue that the method is ready to be used for big and diverse data sets. The authors should increase the test data set notably to deliver these arguments and find a way to quantify the robustness of the analysis pipeline for this bigger data set. Another point is that all organoid lines used in the manuscript are morphologically quite similar: round, cystic, rather homogenous. To argue for robustness in the recognition of different organoid shapes it would be more convincing to add organoid data e.g. of mouse intestinal organoids with recognizable differences in morphology characterized by protrusions, etc.

**RESPONSE 2: We agree with the reviewer with the small size of our test dataset. We also agree that the organoids used in our test dataset had a similar overall morphology. We have addressed this with an expanded the test dataset with 19 images of mouse small intestinal organoids, as suggested by the reviewer. The original OrganoID platform was evaluated with this expanded dataset, and these results are reported in the revised manuscript (page 5):**

*“19 images of organoids derived from mouse small intestine were segmented and evaluated with the model and passed the benchmark with a mean IOU of 0.70 (SD=0.15)”*

**We have also included these results as an additional panel in Figure 1c:**

**The IOU of all of the mouse small intestinal images is also now included in Supplementary Figure 2:**

**We further address the performance on these images in RESPONSE 8 with network retraining.**

The authors segment, track and analyze individual organoids. This might be advantageous for many experimental designs and might moreover reduce the variance of the measured parameters. However, did the authors systematically compare batch analysis and individual (tracked) analysis? Is there a firm advantage of the individual tracking over a batch quantification for some features? Would for example geometrical features be less descriptive or informative at a batch quantification level of time snapshots as compared to individually tracked organoids? Does the variance change indeed?

**RESPONSE 3: We thank the reviewer for recognizing the benefits of organoid tracking in experimental designs for reducing variance in measurements across replicates. We agree that a comparison of “single-organoid snapshot” measurements vs. tracked organoid measurements is important. To address this, we have added an additional analysis of the PDAC organoids in our gemcitabine experiment that compares the variance across replicates when organoid features are normalized to the average organoid measurement at t=0 (batch/snapshot) vs. each individual organoid feature when initially detected by tracking. These results have been included in the new Supplementary Figure 6:**

*Supplementary Figure 6. Comparison of batch vs. tracked normalization. In batch analysis, measurements of organoids exposed to the same gemcitabine dosage for the same duration were normalized to the average of organoids at t=0. In tracked analysis, organoid measurements are instead normalized to each individual organoid measurement when initially detected by the tracking algorithm. The coefficient of variation (CV) was significantly lower with tracked analysis for change in fluorescence (Welch’s t-test p=2e-30), area (p=2e-29) and perimeter (2e-63). CV was significantly higher with tracked analysis for eccentricity (p=7e-25) and circularity (p=2e-12).*

**From these results, there is a significant advantage of the individual tracked approach for reducing variance in features such as organoid area. Other features, such as solidity or eccentricity, did not benefit from tracked normalization. Evidently, organoid size at a given time point is highly dependent on the initial organoid size, while organoid shape shows less dependence. We have clarified this critical point in the text with the following (page 7):**

***“Measurements of death stains such as PI are typically normalized to a viability measurement that accounts for differences in the number and size of organoids between replicates that can compound over the duration of an experiment. We hypothesized that OrganoID could be used to measure morphological features indicative of organoid viability over time, which could then be used to normalize and decrease the variability of measurements across experimental replicates. Furthermore, organoid tracking with OrganoID would enable per-organoid measurement normalization, which we observed to significantly decrease the coefficient of variation of organoid area and fluorescence change across organoids imaged at the same time point and exposed to the same concentration of gemcitabine (Supp. Figure 6).”***

How susceptible is the tracking algorithm for the time interval at which images are taken? For experimental reasons, the time interval might be as low a one or two images per day, would the tracking still work reasonably well and what would be the actual effect on tracking accuracy?

**RESPONSE 4: We have addressed this important question with an additional analysis (numbers are reported in extended data) and sentence in the text (page 7):**

***“Tracking accuracy was not affected when frames were skipped to simulate a 12-hour imaging interval, which demonstrates low susceptibility to imaging frequency.”***

In Figure 3, the effect of gemcitabine should be shown as dose-response curves in addition to the temporal plots. Some of the dose-response curves in Supplementary Figure 5 should appear in the main figure. Does the cell area follow a dose-response curve as well? How does the EC50 measured based on PI or cell area measurements compare to the one determined by the MTS assay; do they match?

**RESPONSE 5: We have created and included a new Figure 3d that includes dose-response curves from the original Supplementary Figure 5. In this new figure, we also have calculated the dose response curves and EC50 for total cell area, total PI intensity, and tracked organoid fluorescence per area. The EC50 for organoid fluorescence per area indeed matches the MTS assay:**

*Figure 3d. An MTS assay was used as a gold standard measurement of viability to compute the half-maximal effective concentration (EC50) at the end of the experiment. Total organoid area, total fluorescence, and area-normalized fluorescence were also measured at endpoint to produce dose-response curves and estimate EC50 without the need of live-cell staining.*

The analysis of cell shape parameters such as circularity, roundness, compactness, solidity, etc is more dependent on accurate pixel segmentation at the object’s circumference than of area. Filtering steps might smoothen the outlines of segmented organoids. How do shape parameters such as circularity compare between automatically and manually segmented organoids? For the analysis of circularity, the authors use phase-contrast microscopy images, whose analysis benefit from sharper contours. Does the shape quantification of brightfield image sources give similar results or are they more error-prone?

_­_**RESPONSE 6: To address this comment, we have added an additional supplementary figure (Supplementary Figure 4) to evaluate performance of organoid shape segmentation and the difference between analysis of phase-contrast and brightfield microscopy images:**

*Supplementary Figure 4. Evaluation of organoid shape measurement. OrganoID was used to measure organoid circularity (ratio of organoid area to the area of a perfect circle with equal perimeter), solidity (ratio of organoid area to area of the convex hull), and eccentricity (elliptical deviation from a circle). These measurements were then compared to those from manual segmentation. The concordance correlation coefficient (CCC) was computed for all organoids, as well as for organoids imaged through phase contrast (PC, red) or brightfield (BF, blue) microscopy. For calculation of CCC for circularity and solidity (bounded to 0-1, with most values near 1), the data was first logit-transformed.*

**We also included these results in the text (page 6):**

***“Several additional morphological metrics were calculated, including circularity (the ratio of the organoid perimeter to the perimeter of a perfect circle), eccentricity (a measure of elliptical deviation from a circle), and solidity (the ratio of the organoid area to the area of its convex hull). These three metrics showed moderate concordance (0.52, 0.41, and 0.59 respectively) between automated and manual measurements (Supp. Figure 4). Performance on brightfield microscopy images was higher than performance on phase-contrast microscopy images.”***

The authors do not explain well the selection of circularity as critical parameter for drug response. Did the authors also analyze other shape parameters in an unbiased way such as the ones mentioned above? I would suggest analyzing several parameters and find the most discriminative parameter. In general, the statement that circularity is a predictive parameter is not convincingly argued. The distribution of circularity and correlation with PI intensity seems minimal and the used statistical one-way ANOVA test to compare a few dozen cells should not be used. Instead, a test used for high effect size such as Cohen’s D should be used to test significance. Circularity should be shown in a dose-response curve.

**RESPONSE 7: We agree with this great suggestion by the reviewer and have investigated two other shape parameters: solidity and eccentricity. We compared OrganoID measurements of these parameters to manual measurements (discussed in RESPONSE 4). We also agree with the reviewer that the ANOVA test should not be used here, and have instead used Cohen’s *d* statistic to measure the effect size of gemcitabine dosage on these shape parameters. The results are now reported in the new Figure 3e:**

*Figure 3e. Descriptive shape measurements, including circularity (the inverse ratio of organoid perimeter to the perimeter of a perfect circle with equivalent area), solidity (the ratio of organoid area to its convex hull), and eccentricity (elliptical deviation from a perfect circle), were computed for each organoid. The effect size (Cohen’s d statistic) between each gemcitabine concentration is shown. Effect size is computed from dosages on the x-axis to dosages on the y-axis.*

**We also have computed dose-response curves for more organoid shape parameters as suggested by the reviewer: circularity, solidity, and eccentricity, and included them in new supplementary figure 7. These values followed sigmoidal responses with reasonable EC50s:**

*Supplementary Figure 7. Dose response of shape metrics. Organoid circularity, solidity, and eccentricity were observed to follow sigmoidal dose responses to gemcitabine.*

**These new results have also been reported in the revised text (page 8):**

***“Organoid circularity, solidity, and eccentricity showed sigmoidal dose responses and determined the endpoint EC50 to be 28.47 nM, 27.05 nM, and 18.08 nM, respectively (Supp. Figure 7). The effect size of gemcitabine concentration on these shape metrics was also computed with Cohen’s d statistic, defined as the ratio of the difference in means to the pooled standard deviation. Gemcitabine dosage had a moderate to large effect on organoid circularity, solidity, and eccentricity across concentrations below and above the EC50 (Figure 3e). Circularity and solidity decreased at higher concentrations, while eccentricity increased, reflecting disrupted organoid morphology”***

Lastly, from the perspective of a potential user it would be interesting to know if the neuronal network can be readily extended by user-owned imaging data to improve the segmentation outcome and compensate for experimental variation due to differences in the specific equipment, imaging devices, etc. In case this can be done, it could be brought up in the discussion.

**RESPONSE 8: We thank the reviewer for their interest in our platform as a potential user. The network can indeed be extended by user-owned imaging data. To address this in our publication we have now demonstrated continued retraining of the OrganoID platform on new “user-owned” images. As mentioned in RESPONSE 2, we evaluated the network on a new image dataset of mouse small intestinal organoids. While *mean* performance on this dataset passed our benchmark (IOU of 0.70), some images were below the threshold. To improve performance on this dataset, we resumed training of the model with the mouse organoid images added in. The instructions for how to do this are included in the OrganoID repository (**[**http://www.github.com/jono-m/OrganoID.git**](http://www.github.com/jono-m/OrganoID.git)**). The results of the retrained model have been incorporated into new Supplementary Figure 2, below the performance of the original model:**

**This has also been now described in the revised text (page 5):**

***“Additionally, 19 images of organoids derived from mouse small intestine were segmented and evaluated with the model and passed the benchmark with a mean IOU of 0.70 (SD=0.15). These results support the potential of the OrganoID neural network to generalize to organoids from various tissues of origin without parameter tuning (Figure 1d). However, 3 images of mouse small intestinal organoids did not pass the benchmark, which suggests a limit to this generalizability. To demonstrate how performance can be improved on novel datasets, a copy of the neural network was additionally trained on a subset of mouse small intestinal organoid images. This retrained network showed considerable improvement on a test dataset of mouse intestinal organoids with a mean IOU of 0.82 (SD=0.09) while maintaining performance on the original test dataset with a mean IOU of 0.76 (SD=0.10).”***

Reviewer 2

Heterogeneity in organoid morphology complicates high throughput, automated image analysis of multiple organoids growing within a substrate. Preparation of images for analysis by imaging software requires manual adjustment of focal plane and some “eyes-on” interpretation for each image is necessary. The authors have developed software that can monitor individual organoids representing any tissue type and provide morphometric data. The major accomplishment is the ability to distinguish multiple organoids within a cluster, and track and provide data on each. The strategy is label-free and non-destructive, enabling longitudinal studies. Overall, the manuscript is clear, concise, and well written. Impact is judged to be high, based on the tremendous need for automated tracking and characterization of individual human tissue derived organoids within a population of such organoids. The technique described leverages artificial intelligence technology, of which, this reviewer has no specific expertise. However, the challenges of automated differentiation and tracking of overlapping, clonally expanding cell populations in microscopic images is well understood by many of us in the 3D in vitro modeling community.

**RESPONSE 9: We thank the reviewer for acknowledging the challenge of image analysis for experiments with 3D tissue models and the powerful advantages that OrganoID provides for organoid studies. We also thank the reviewer for the positive feedback on the quality of our manuscript and impact of this work.**

Major strengths:

- Validation of an AI algorithm for discriminating, and tracking individual organoids within a population, over time, and in a label-free manner.
- Ability to integrate morphometric analyses that provide some degree of phenotypic characterization. These capabilities were demonstrated through studies involving the determination of chemotherapeutic agent effectiveness on a pancreatic tumor organoid population. These studies did include propidium iodide, as training data. These experiments were then extended to label-free evaluation of organoid health in label-free studies. It is obvious that further algorithm training may expand significantly on these capabilities.
- Overall, the manuscript is clear, concise, and well written. It was a pleasure to read and would be easily understood by someone with either a computer science or biomedical background.
- Impact is judged to be high, based on the tremendous need for automated tracking and characterization of individual human tissue derived organoids within a population of such organoids.

**RESPONSE 10: We again thank the reviewer for their positive comments regarding our research. We agree that additional training of the neural network can considerably improve results. To address this, we have now extended OrganoID to mouse small intestinal organoids, which is shown in the revised Figure 1c:**

**The new mouse organoid image dataset was also used to additionally train the model, and performance is also reported in the revised Supplementary Figure 2 along with the performance of the original model:**

Minor criticism

As stated above, the manuscript is easily followed by a reader with a life sciences background. That being said, this reviewer was forced to Google several terms to understand the significance. A few extra sentences would have made this task unnecessary for grasping the significance of sentences such as, “We took advantage of this phenomenon to identify and separate organoid contours with a modified Canny edge detector (to define organoid boarders) and a watershed transformation (to differentiate individual organoid within an overlapping cluster).

**RESPONSE 11: This comment highlighted that we should clarify particular terms that should not be assumed as prior knowledge from the reader. We have revised our manuscript accordingly.**

**For this specific example, we changed the text to (page 6):**

***“We took advantage of this phenomenon to identify organoid contours with a Canny edge detector^30^, an image transformation which (i) computes the partial derivative of pixel intensity to identify high-contrast areas, (ii) blurs the image to smooth noisy regions, and (iii) applies a hysteresis-based threshold to identify locally strong edges. Edges are removed from the thresholded prediction image to mark the centers of each organoid. These centers are then used as initializer basins in a watershed transformation^31^, an algorithm inspired by the filling of geological drainage basins to separate touching objects.”***

***Other relevant changes we made are shown with tracked changes in the revised manuscript.***

In summary, the manuscript describes an AI algorithm that can differentiate and track multiple individual organoids within a population that has a significant degree of overlap. This capability may be further enhanced through continued training that may provide phenotypic characterization based on morphometric parameters. The information is expertly communicated and the impact for this work is deemed high.

**RESPONSE 12: We again thank the reviewer for their helpful feedback and appreciate the positive comments about the impact of this research.**

Reviewer 3

In this manuscript, the authors present their findings of a machine-learning-based image analysis model, OrganoID. The authors have developed a novel platform in OrganoID that can be used to monitor drug responses by tracking organoid dynamics via deep learning. The authors have emphasized the importance of studying organoids, highlighted the limitations of the existing approach, and clearly stated the motivation for an automated image analysis tool. Furthermore, the greatest strength of this model is that it can be applied to a variety of tissues. Overall, the paper is very well written especially the results section which has been broken down under further subheadings to make the paper easily comprehensible. A few revisions and clarifications are recommended to make the paper more robust.

**RESPONSE 13: We thank the reviewer for recognizing the advantages of OrganoID for analysis of multiple organoid types and for the positive comments about the results section. We appreciate the reviewer’s insightful feedback.**

“Three-dimensional (3D) cell culture systems serve as more physiologically relevant in vitro models than traditional human cell monolayers for both basic and clinical applications” (Why is that so? It is suggested that the authors further elaborate and expand on this claim. Furthermore, what do the authors mean by the phrase “physiologically relevant” - the reference cited states that organoid provides a powerful means for “organogenesis” and “tissue morphogenesis” – is that what the authors are referring to?)

**RESPONSE 14: We have restructured this paragraph to address this comment and removed the phrase “physiologically relevant” for a clearer statement (page 2):**

***“Organoids closely recapitulate cellular heterogeneity, structural morphology, and organ-specific functions of a variety of tissues, which improves modeling of in situ biological phenomena compared to traditional monolayer cell cultures.”***

“Live-cell imaging can reveal important features of organoid dynamics, such as growth, apoptosis/necrosis, and movement, which can reflect physiological and pathological behaviors and drug responses.” (Please cite a reference for this statement. What are authors referring to specifically when they are talking about “physiological and pathological behaviors”?)

**RESPONSE 15: We have added citations and regrouped the inline citations for the relevant sources to correspond to this statement. The statement has also been modified to (page 2):**

***“Live-cell imaging can reveal important features of organoid dynamics, such as growth, apoptosis/necrosis, movement, and drug responses, which can reflect physiological and pathological events such as organ development, function, infarction, cancer, and infection.”***

**There are six cited examples that we now provide for this statement:**

*Driehuis, E. et al. Oral Mucosal Organoids as a Potential Platform for Personalized Cancer Therapy. Cancer Discovery 9, 852–871 (2019).*

*Dutta, D., Heo, I. & Clevers, H. Disease Modeling in Stem Cell-Derived 3D Organoid Systems. Trends in Molecular Medicine 23, 393–410 (2017).*

*Sachs, N. et al. A Living Biobank of Breast Cancer Organoids Captures Disease Heterogeneity. Cell 172, 373-386.e10 (2018).*

*Clevers, H. & Tuveson, D. A. Organoid Models for Cancer Research. Annual Review of Cancer Biology 3, 223–234 (2019).*

*van der Vaart, J. et al. Modelling of primary ciliary dyskinesia using patient‐derived airway organoids. EMBO Rep 22, (2021).*

*Richards, D. J. et al. Human cardiac organoids for the modelling of myocardial infarction and drug cardiotoxicity. Nat Biomed Eng 4, 446–462 (2020).*

“While organoids have been successfully used to investigate important phenomena that might be obscured in simpler models, their use in data-intensive applications, such as high-throughput screening, has been more difficult.” (Please cite a reference for this claim. Furthermore, it is suggested that the authors expand further on why it has been more difficult)

**RESPONSE 16: For clarity and to address this comment, we have moved this sentence to the start of the next paragraph, which now describes in-depth the challenges of using organoids for high-throughput screening. We also added the relevant citations.**

“The OrganoID neural network follows a u-net structure and was optimized to require far fewer feature channels than the original implementation.” (It is suggested that the authors explain why they decided to optimize it to require fewer channels for clarity)

**RESPONSE 17: We have added on to this sentence with an explanation of our decision to optimize the model (page 4):**

***“The OrganoID neural network follows the u-net structure and was optimized to require far fewer feature channels than the original implementation, as a reduction in trainable model parameters limits overfitting and minimizes the amount of memory and computational power required to use the network in the final distribution.”***

“Model simplification can limit overfitting and minimizes the amount of memory and computational power required to use the network in the final distribution.” (Is this statement made by the authors based on the results of prior study if yes, please cite the relevant study; however, if this is based on the authors' experiences it is suggested that they explain this further.)

**RESPONSE 18: We have added a citation for this statement:**

***Bejani, M.M., Ghatee, M. A systematic review on overfitting control in shallow and deep neural networks. Artif Intell Rev 54, 6391–6438 (2021). https://doi.org/10.1007/s10462-021-09975-1***

“Additionally, all hidden convolutional layers were set to compute outputs with the exponential linear unit (ELU) activation function26, which has demonstrated higher accuracy than the rectified linear unit (ReLU) and avoids vanishing gradient problems during network training.” (Did it demonstrate a higher accuracy during this specific study’s network training or during another study – please elaborate and add specific statistics to highlight the “higher accuracy”. It would be helpful to back the replacement of ELU with ReLU with IOU scores)

**RESPONSE 19: This statement on higher accuracy comes from the original ELU publication cited, and has been reworded to reflect this more clearly in the revised manuscript (page 4):**

***“…which has been previously shown to produce higher accuracy than the rectified linear unit (ReLU, used in the original u-net implementation) and avoid vanishing gradient problems during network training^28^”***

***28	Clevert, D.-A., Unterthiner, T. & Hochreiter, S. Fast and Accurate Deep Network Learning by Exponential Linear Units (ELUs). (2015) doi:10.48550/ARXIV.1511.07289.***

“18 microscopy images of organoids derived from lung epithelia, colon epithelia, and salivary adenoid cystic carcinoma (ACC) were manually segmented.” (Were they 18 images from each tissue or 18 combined? If 18 combined please highlight how many images were used from each tissue. Why were such few images available?)

**RESPONSE 20: We have clarified the image breakdown in the text (page 5):**

***“Microscopy images of organoids derived from lung epithelia, colon epithelia, and salivary adenoid cystic carcinoma (ACC) were manually segmented (6 images for each tissue type).”***

**These images had to be hand-segmented pixel-by-pixel and contained hundreds of organoids, and we had limited time to expand the dataset further (however our measurements have all been reported with the appropriate standard deviations). We added 19 images of mouse intestinal organoids to the test dataset in this revision.**

“The OrganoID network passed the benchmark for all non-PDAC images, with a mean IOU of 0.79 (SD=0.096). These results demonstrate the capability of the OrganoID neural network to reliably detect organoids from various tissues of origin, from both malignant and non-malignant tissues, in a pixel-by-pixel manner “(An IOU greater than 0.5 definitely demonstrates the capability of the model but perhaps saying the results are “reliable” without any statistical test to back it up is misleading. Given the small sample size may be the authors should revise this statement. In the introduction section, the authors have shared the relevant limitations of existing approaches in the literature. However, if feasible, it will be helpful to report the performance (IOU) of existing/ old approaches for at least semantic segmentation tasks to serve as a good reference point for comparing the proposed architecture.)

**RESPONSE 21: We have added citations to compare performance of our model to models used for cell nuclei segmentation:**

***Englbrecht, F., Ruider, I. E. & Bausch, A. R. Automatic image annotation for fluorescent cell nuclei segmentation. PLoS ONE 16, e0250093 (2021).***

***Long, F. Microscopy cell nuclei segmentation with enhanced U-Net. BMC Bioinformatics 21, 8 (2020).***

**We have also added 19 images of organoids derived from mouse small intestine to the analysis, and revised the statement for clarity (page 5):**

***“The OrganoID network passed the benchmark for all of these non-PDAC images, with a mean IOU of 0.79 (SD=0.096). Additionally, 19 images of organoids derived from mouse small intestine were segmented and evaluated with the model and passed the benchmark with a mean IOU of 0.70 (SD=0.15). These results support the potential of the OrganoID neural network to generalize to organoids from various tissues of origin without parameter tuning (Figure 1d).”***

“For quantitative validation, OrganoID was used to count and measure the area of organoids in images from the PDAC and non-PDAC testing datasets (a total of 28 images).” (Why was the analysis not conducted on a larger image dataset?)

**RESPONSE 22: As mentioned in RESPONSE 20 and RESPONSE 21, these images had to be hand-segmented pixel-by-pixel and contained hundreds of organoids, and we had limited time to expand the dataset further (however our measurements have all been reported with the appropriate standard deviations). To address this concern, we increased the size of our image dataset with new 19 images of mouse intestinal organoids to the test dataset in this revision.**

“Overall, the measurements produced by OrganoID were in considerable agreement with those obtained by hand, which supports the use of OrganoID for automated single-organoid analysis.” (Yes, the results demonstrate considerable agreement between the two however, the image dataset size is too small to claim that the results support the use of OrganoID for automated single-organoid analysis. Please revise this statement perhaps instead to state that it demonstrates that the model is capable of identifying organoids when compared to hand however, to demonstrate its utility it needs to be trained on more data.)

**RESPONSE 23: We have added to this statement to reiterate that this analysis is only on our test dataset (page 6):**

***“Overall, the measurements produced by OrganoID were in considerable agreement with those obtained by hand in our test dataset, which supports the potential use of OrganoID for automated single-organoid analysis.”***

**We have also added a worked example of retraining the network on images of mouse small intestinal images, which demonstrates to the reader that our model is by no means perfect and that utility can always be improved with continued training on more data. We included the following statement in the revised manuscript” (page 5)**

***“However, 3 images of mouse small intestinal organoids did not pass the benchmark, which suggests a limit to this generalizability. To demonstrate how performance can be improved on novel datasets, a copy of the neural network was additionally trained on a subset of mouse small intestinal organoid images. This retrained network showed considerable improvement on a test dataset of mouse intestinal organoids with a mean IOU of 0.82 (SD=0.09) while maintaining performance on the original test dataset with a mean IOU of 0.76 (SD=0.10).”***

“Identification of individual organoids with diverse morphology and size” (The edge prediction depends on the labeling protocol used for the data with a thin boundary between organoids. This might not be true for another organoid dataset. It will be helpful if the authors can add some points for expanding this approach to a new dataset.)

**RESPONSE 24:** **We have added to our description of the thin boundary (page 5-6):**

***“The segmentations that were used to train the neural network were produced with a 2-pixel boundary between organoids in contact that follows the contour of separation.”***

**We have also released the OrganoID platform with all training and testing images and segmentations for reference by users. In particular, we have included a worked example of expanding our approach to an image dataset of mouse small intestinal organoids (**[**http://github.com/jono-m/OrganoID.git**](http://github.com/jono-m/OrganoID.git)**).**

The tracking step was also performed by hand to evaluate automated performance, which maintained over 89% accuracy throughout the duration of the experiment (What statistical test was used to measure this accuracy?)

**RESPONSE 25: We have added a clear definition for accuracy for this result:**

***“OrganoID tracking maintained over 89% accuracy (defined as the fraction of identified organoids that were correctly matched at each time step) throughout the duration of the experiment.”***

**We would like to note that this accuracy was measured with an N-of-1 timelapse experiment and , as such, we have not made any statements on the statistical significance of this result.**

Each organoid shape was then used to calculate the organoid circularity, a morphological measurement that compares the organoid perimeter to the perimeter of a perfect circle. (Given the morphology of the cells it is understood why this parameter was used but perhaps the authors should go into more detail about why they picked organoid circularity and why they think it is related to overall cellular architecture.)

**RESPONSE 26: To address this, we have added analysis of other morphological features including solidity and eccentricity, which are reported in the reworked Figure 3e:**

**The following statement has also been added to the results section (page 8):**

***“Circularity and solidity decreased at higher concentrations, while eccentricity increased, reflecting disrupted organoid morphology.”***

**The following statements have also been added to the discussion along with a relevant citation (page 9):**

***“In the example gemcitabine dose-response experiment, we observed that PDAC organoid circularity and solidity decreased with an increase in gemcitabine dosage. This disturbance of organoid architecture is likely due to the interference of gemcitabine with RNA and DNA synthesis, which may in turn affect cell turnover and production of signaling and structural proteins.***

***Mini, E., Nobili, S., Caciagli, B., Landini, I. & Mazzei, T. Cellular pharmacology of gemcitabine. Annals of Oncology 17, v7–v12 (2006).”***

“Experimental replicates in organoid studies can differ in the number and distribution of sizes of organoids.” (Given these circumstances how would the authors suggest validating their model?)

**RESPONSE 27: We have used our approach to decrease variance in measured parameters across replicates. These results were presented in the original text, and we have modified the following sentence to clarity (page 7-8):**

***“Normalization of PI intensity with tracked single-organoid area increased the separation of responses between each treatment group over time, decreased standard error across replicates, and corrected the response discrepancy between the 100 nM and 1000 nM conditions.”***

“OrganoID achieves comprehensive and expedient image analysis of organoid experiments to enable the broader use of organoids as tissue models for high-throughput investigations into physiological and pathological phenomena.” (What do the authors mean by physiological and pathological phenomena, please elaborate.)

**RESPONSE 28: This statement has been reworked for clarity, and now reads (page 10):**

***“OrganoID achieves comprehensive and expedient image analysis of organoid experiments to enable the broader use of organoids as tissue models for high-throughput investigations into biological systems.”***

**We also revised a statement in the introduction for the use of organoids as models for biological systems (page 2):**

***“Live-cell imaging can reveal important features of organoid dynamics, such as growth, apoptosis/necrosis, movement, and drug responses, which can reflect physiological and pathological events such as organ development, function, infarction, cancer, and infection.”***

**There are six cited examples that we now provide for this statement:**

*Driehuis, E. et al. Oral Mucosal Organoids as a Potential Platform for Personalized Cancer Therapy. Cancer Discovery 9, 852–871 (2019).*

*Dutta, D., Heo, I. & Clevers, H. Disease Modeling in Stem Cell-Derived 3D Organoid Systems. Trends in Molecular Medicine 23, 393–410 (2017).*

*Sachs, N. et al. A Living Biobank of Breast Cancer Organoids Captures Disease Heterogeneity. Cell 172, 373-386.e10 (2018).*

*Clevers, H. & Tuveson, D. A. Organoid Models for Cancer Research. Annual Review of Cancer Biology 3, 223–234 (2019).*

*van der Vaart, J. et al. Modelling of primary ciliary dyskinesia using patient‐derived airway organoids. EMBO Rep 22, (2021).*

*Richards, D. J. et al. Human cardiac organoids for the modelling of myocardial infarction and drug cardiotoxicity. Nat Biomed Eng 4, 446–462 (2020).*

The methods section mentions that the appropriate number of filters in the first layer has been determined using validation set scores. It will be helpful if the authors can report those numbers.

**RESPONSE 29: We have now reported the requested data in the methods section:**

***“We sequentially reduced the number of filters in the first layer by powers of two to reach a minimal value that preserved performance on the validation dataset (validation mean IOU was 0.088 for 8 filters in the first layer, compared to 0.098 for 4 filters and 0.1 for 16 filters).”***

“OrganoID was trained with and tested on a diverse, but relatively small set of images.” (“a” is missing before relatively)

**RESPONSE 30: This sentence has been changed to the following (page 9):**

**“OrganoID was trained with and tested on a diverse yet relatively small set of images.”**

“Dynamic changes in individual organoid morphology, number, and size can indicate important drug responses, however these metrics are difficult and labor-intensive to obtain for high-throughput image datasets.” (please add a comma after however)

“Three-dimensional (3D) cell culture systems serve as more physiologically relevant in vitro models than traditional human cell monolayers for both basic and clinical applications1. Organoids are multicellular 3D structures derived from primary or stem cells that are embedded into a biological matrix to create an extracellular environment that provides structural support and key growth factors.” (The reviewer suggests that the paragraph should be rephrased such that the concept of organoids is introduced before talking about its basic and clinical applications i.e. “Organoids are multicellular 3D structures derived from primary or stem cells that are embedded into a biological matrix to create an extracellular environment that provides structural support and key growth factors. Three-dimensional (3D) cell culture systems serve as more physiologically relevant in vitro models than traditional human cell monolayers for both basic and clinical applications”)

Many of these existing platforms were also developed for analysis of organoids derived from a single type of tissue and for images obtained with one specific geometric configuration, precluding their use across different experimental settings. (“the” missing – many of these existing platforms were also developed for “the” analysis of organoids)

“The image is further refined to remove organoids that may be partially out of the field-of-view or are below a particular size threshold.” (The dashes between “field-of-view”are not needed)

“We also compared these normalized measurements to normalization with an MTS proliferation assay, which can only be used for endpoint analysis.” (what does MTS stand for?)

“…express fluorescent proteins, however this increases experimental time and complexity and may alter cellular dynamics from the original tissue sample.” (add a comma after however)

**RESPONSE 31: We thank the reviewer for close proofreading! All of these suggested grammatical corrections have been made. The full name for MTS has been added.**
